# A survey of preparedness against coronavirus disease 2019 (COVID-19) in hospitals in Tokyo, Japan, with healthcare personnel with COVID-19 and in-facility transmission

**DOI:** 10.1017/ice.2020.1304

**Published:** 2020-10-29

**Authors:** Yasuaki Tagashira, Akane Takamatsu, Shinya Hasegawa, Yuki Uenoyama, Hitoshi Honda

**Affiliations:** 1 Division of Infectious Diseases, Tokyo Metropolitan Tama Medical Center, Tokyo, Japan; 2 Department of Microbiology, Juntendo University Graduate School of Medicine, Tokyo, Japan

## Abstract

A questionnaire was distributed to hospitals in Tokyo (N = 38) regarding their preparedness against and in-facility transmission of coronavirus disease 2019 (COVID-19). As of May 31, 2020, 284 HCP had contracted COVID-19, and in-facility COVID-19 transmission occurred at 13 hospitals, negatively impacting hospital functions and patient care.

Healthcare personnel (HCP) are at much higher risk than the general population of contracting coronavirus disease 2019 (COVID-19), despite advanced hospital infection control strategies.^1^ The Centers for Disease Control and Prevention in the United States reported that ~500 frontline HCP in the United States had died from COVID-19 by the end of July 2020.^2^ In the present study, we used an Internet search to identify hospitals in Tokyo that issued press releases or held press conferences about their HCP who contracted COVID-19. Questionnaires were then distributed to these hospitals regarding their COVID-19 pandemic preparedness, routes of HCP infection, and resulting impacts on patient care and hospital functions.

## Methods

### Study design

The present study was based on responses to a questionnaire sent to hospitals in Tokyo, Japan, regarding HCP with COVID-19.^3^ The cumulative number of patients with laboratory-confirmed COVID-19 in Tokyo at the time of this study was 5,236.^4^ We searched the websites of healthcare facilities that issued press releases or held press conferences regarding their HCP with COVID-19 between January 1, 2020, and May 31, 2020. We also searched the Internet for news about hospitals with infected HCP using the same key words. Overall, we identified 53 hospitals. The survey was conducted from June 4 to 17, 2020. The questionnaire collected the following information: (1) probable COVID-19 transmission routes and outcomes, including clinical outcomes, in all the infected HCP and patients; (2) nosocomial transmission to inpatients and their clinical outcomes; (3) impact of COVID-19 transmission on hospital functions; (4) institutional preparedness against COVID-19; and (5) deleterious effects on hospital function related to the presence of infected HCP and nosocomial COVID-19 transmission. Nosocomial transmission was defined as in-facility COVID-19 onset, whereas community acquisition was defined as all other infections. The onset of infection was determined solely by the respondents.

## Results

### Baseline information on hospitals with HCP with COVID-19

Overall, 53 hospitals with HCP with COVID-19 were identified based on press releases, press conferences, or media reports. Among these, 38 (72%) participated in our survey. Appendix 1 (online) shows their baseline characteristics.

In total, 284 HCP in 38 hospitals contracted COVID-19. The median age of the infected HCP was 32 years (range, 20–81), and 211 (74%) were female. Nurses accounted for 141 cases, and 155 personnel (55%) were involved in caring for patients with COVID-19 before becoming infected. Although 22 of 38 (58%) of the first HCP to become infected at each participating hospital were thought to have acquired the disease via community-onset infection, a large proportion of supervenient infections among HCP was thought to be due to nosocomial transmission (Table 1 and Appendix 2 online). Although 267 of these HCP (94%) subsequently returned to work, 12 (4%) were suspended or retired after contracting COVID-19.

**Table 1.** Details of Institutional Experiences for HCP and Patients With COVID-19

Characteristics	No. (%) (N=38)
HCP with COVID-19	
No. of HCP with COVID-19, median (range)	3 (1–51)
Duration of suspension from work in infected HCP or those with close contact to patients with COVID-19, median d (range)	14 (5–14)
**Mode of initial transmission in HCP^a^**	
Community acquisition	22 (57.9)
In-facility (nosocomial) transmission	10 (26.3)
Unknown	6 (15.8)
**Preventive measures for HCP in close contact with COVID-19 patients**	
None	1 (2.6)
Home quarantine only	5 (13.2)
Home quarantine + PCR for all HCP in close contact with COVID-19 patients	20 (52.6)
Home quarantine + PCR for symptomatic HCP in close contact with COVID-19 patients	3 (7.9)
Continued work + PCR for all HCP in close contact with COVID-19 patients	7 (18.4)
Continued work + PCR for symptomatic HCP in close contact with COVID-19 patients	2 (5.3)
Cumulative number of PCR tests for HCP as of May 31, 2020 median (range)	53 (2–697)
In-facility (nosocomial) transmission^a^	
In-facility (nosocomial) transmission to hospitalized patients	13 (34.2)
No. of hospitalized patients contracting COVID-19 via in-facility (nosocomial) transmission, average, (range)	4 (0–37)
Impact of in-facility (nosocomial) transmission on hospital function	
**Outpatient function**	
None	15 (39.5)
Nonadmission of new outpatients	16 (42.1)
Restricted follow-up visits	7 (18.4)
Nonadmission of new patients in emergency department (n=34)	15/34 (44.1)
**Inpatient function**	
None	10 (26.3)
Nonadmission of new inpatients in all inpatient wards	11 (28.9)
Nonadmission of new inpatients in wards with in-facility (nosocomial) transmission	12 (31.6)
Closure of inpatient wards with in-facility (nosocomial) transmission	10 (26.3)
Closure of all inpatient wards	1 (2.6)
**Disclosure of HCP with COVID-19 or in-facility (nosocomial) transmission of COVID-19**	
Website only	28 (73.7)
Press conference only	1 (2.6)
Website + press conference	2 (5.3)
Website + press release	7 (18.4)
Media coverage of HCP with COVID-19 or in-facility (nosocomial) transmission	23 (60.5)
Request for interviews regarding HCP with COVID-19 or in-facility (nosocomial) transmission by mass media	8 (21.1)
Negative effects on HCP with COVID-19 or in-facility (nosocomial) transmission	
Harassment or stigmatization	18 (47.4)
**Media reporting on HCP with COVID-19 or in-facility (nosocomial) transmission at study center**	
Critical	8 (21.1)
Neutral	18 (47.4)
No reporting	11 (28.9)
**Media reporting on HCP with COVID-19 or in-facility (nosocomial) transmission at other hospitals**	
Critical	24 (63.2)
Neutral	14 (36.8)
**Reaction of patients to HCP with COVID-19 or in-facility (nosocomial) transmission**	
Critical	9 (23.7)
Neutral	21 (55.3)
Supportive	8 (21.1)
**Challenges in infection control practice during COVID-19 pandemic**	
Inadequate hospital infrastructure	31 (81.6)
Suboptimal PCR testing strategies (eg, testing capacity, access)	26 (68.4)
Suboptimal computerized operating system (eg, electronic reports)	11 (28.9)
Shortage of personal protective equipment	32 (84.2)
Shortage of alcohol-based hand hygiene products	21 (55.3)
Lack of financial support	16 (42.1)
Lack of hospital leadership in infection control practice	10 (26.3)
Lack of cooperation and support from local department of health	6 (15.8)
Insufficient number of HCP engaged in infection control	23 (60.5)
Lack of financial or welfare support for affected HCP	12 (31.6)
Lack of cooperation among HCP not directly involved in COVID-19 care	18 (47.4)

Note. HCP, healthcare personnel; COVID-19, coronavirus disease 2019.

a See ‘Methods’ for definition.

### Impacts of COVID-19 transmission on HCP, inpatients, and hospital functions

Table 1 shows the details of the institutional experiences of healthcare personnel and patients with COVID-19. Nosocomial severe acute respiratory coronavirus virus 2 (SARS-CoV-2) transmission to inpatients occurred at 13 of the 38 hospitals (34%). The total number of cases of nosocomial SARS-CoV-2 transmission to inpatients at the 13 hospitals reached 156, and 42 of these patients (27%) died during hospitalization. Of the remaining 114 patients, only 21 of 156 (14%) fully recovered, and 93 of 156 (60%) continued to be hospitalized at the time of data collection.

At most of the hospitals, HCP with suspected exposure were removed from the front line until their negativity for SARS-CoV-2 was confirmed, and HCP with suspected exposure in outpatient services (23 of 38, 61%) and inpatient services (28 of 38, 74%) were restricted or temporarily suspended. In addition, 18 hospitals (47%) reported verbal abuse against both infected and noninfected HCP and their family members following reports of nosocomial SARS-CoV-2 transmission (Appendix 2 online). Moreover, a portion of the respondents felt that media reports negatively affected the functioning of their own hospital (8 of 38, 21%) or other hospitals (24 of 38, 63%).

### Hospital preparedness for COVID-19 and association with in-facility COVID-19 transmission

Table 2 compares preparedness for COVID-19 by the end of February 2020 among hospitals with or without nosocomial COVID-19 transmission. Hospitals with nosocomial transmission may have had less intensive hospital-level infection control practices, such as providing separate passages for patients with suspected COVID-19, or they may not have had a preparedness planning committee or hospital-specific COVID-19 guidance in place by the end of February 2020.

**Table 2.** Hospital Preparedness Against Nosocomial Transmission of COVID-19^a^

Preparedness Achieved by the End of February 2020	All	Hospitals With Nosocomial Transmission (n=13), No. (%)	Hospitals Without Nosocomial Transmission (n=25), No. (%)	*P* Value
Organization of preparedness planning committee	38	6 (46.2)	19 (64.0)	.08
Hospital-specific COVID-19 preparedness guidance (eg, hospital guidelines, manuals, etc) available to all HCP	38	6 (46.2)	22 (88.0)	.02
Creation of separate passages for patients with suspected COVID-19 to minimize cross contamination in emergency department	34	2/11 (18.2)	17/23 (73.9)	.004
Creation of separate passages for patients with suspected COVID-19 to minimize cross contamination in outpatient clinic	38	5 (26.3)	17 (73.9)	.01
Creation of separate passages for patients with suspected COVID-19 for admission to inpatient wards	38	3(23.1)	12(48.0)	.10
Restriction of visits to hospitalized patients	38	9 (69.2)	16 (64.0)	1.00
Universal masking for all hospital visitors	38	8 (61.5)	13 (52.0)	1.00
Universal masking for all HCP	38	11 (84.6)	15 (60.0)	.50
Designation of special wards for patients with COVID-19	38	4 (30.8)	10 (40.0)	.73
Hospital-wide symptom reporting system for HCP	38	8 (61.5)	12 (48.0)	1.00

Note. SARS-CoV-2, severe acute respiratory syndrome coronavirus 2; COVID-19, coronavirus disease 2019; HCP, healthcare personnel.

a Facilities with nosocomial transmission were reported by the respondents.

## Discussion

By May 31, 2020, several hospitals in Tokyo reported nosocomial transmission of COVID-19 and infected HCP. As elsewhere, COVID-19 infections were common among HCP, who are at high risk of contracting the disease during patient treatment.^5–7^ Apparently, a common route of initial transmission among the HCP at each hospital was community rather than nosocomial transmission, with the remaining cases likely due to nosocomial transmission. In the early phase of the pandemic, close contact with patients with COVID-19 during routine patient care may have led to HCP infections, which might then have been transmitted through sharing common spaces during break times.^8^ Asymptomatic or minimally symptomatic cases among HCP can also result in transmission.^9^ Nosocomial transmission can also be exacerbated by shortages of personal protective equipment and alcohol-based sanitizers.^10,11^

Unfortunately, a high mortality rate among inpatients with COVID-19 due to nosocomial transmission was observed, in line with other reports.^12^ The findings of the present study underscored the importance of preventing the nosocomial transmission of COVID-19.

Moreover, the emergence of infections among HCP and the nosocomial transmission of COVID-19 further deleteriously affected patient care and hospital functions by producing various negative social effects, including verbal abuse of HCP by the public, criticism by the media, and negative patient responses (Appendix 3 online). Verbal abuse of infected HCP has been reported globally.^13–15^ Changes in the public mindset and improved understanding of COVID-19 transmissibility are needed to eliminate this sort of behavior.^14^

In general, inadequate hospital preparedness during the early phase of the pandemic was likely associated with nosocomial COVID-19 transmission. The community spread of COVID-19 began in February 2020, and hospital preparedness by the end of February 2020 serves as an important index of the preparedness mindset prior to the surge in infections. The preparedness planning and organization of committees at the hospitals in this study demonstrated the generally proactive efforts of the hospital leadership. The availability of hospital-wide guidelines to deal with COVID-19 and prompt implementation of infection control practices reflected the general competence of the infection control departments. Moreover, other hospital-wide measures, such as providing special passages for patients with suspected COVID-19 to minimize potential facility-wide contamination, were more common among hospitals without nosocomial transmission.

This study has several limitations. The exact mode of transmission to either HCP or inpatients was unable to be determined due to the lack of a clear definition of nosocomial transmission and community-onset COVID-19 infection. Because the determinations of the transmission mode and infection onset were made by individual infection control personnel and were self-reported by the facility, the data were not validated. Moreover, because the questionnaire depended completely on the respondents’ impression and reports, a response bias may have been introduced. Finally, our study focused solely on hospitals in Tokyo; thus, the findings may not reflect the situation elsewhere.

In conclusion, COVID-19 transmission among HCP and nosocomial transmission to inpatients was common and negatively affected hospital functioning and patient care. Furthermore, to preserve the safety and dignity of HCP with COVID-19, changes in the public’s mindset and better understanding of the transmissibility of COVID-19 are needed.

## Supplementary material

For supplementary material accompanying this paper visit http://dx.doi.org/10.1017/ice.2020.1304. click here to view supplementary material
