# Supplementary material for: A survey of preparedness against coronavirus disease 2019 (COVID-19) in hospitals in Tokyo, Japan, with healthcare personnel with COVID-19 and in-facility transmission
Source: Infect Control Hosp Epidemiol. 2020 Oct 29:1–5. doi: 10.1017/ice.2020.1304 (PMC7684020; doi:10.1017/ice.2020.1304)
Supplement: Supplementary file 1 [file S0899823X20013045sup001.docx]

Appendix 1. Baseline characteristics of hospitals reporting HCP with COVID-19

| Characteristics | N=38 |
| --- | --- |
| Hospital type |  |
| University-based hospital | 5 (13.2) |
| University-affiliated hospital | 3 (7.9) |
| Public hospital | 7 (18.4) |
| Private hospital | 23 (60.5) |
| Hospital type by function* |  |
| Specialty hospital | 7 (18.4) |
| General hospital | 22 (60.5) |
| Regional medical care support hospital | 8 (21.1) |
| Long-term care facility | 1 (2.6) |
| Hospitals designated by the Japanese government for the treatment and containment of specific infectious diseases. | 9 (23.7) |
| Number of beds, median (range) | 398 (41-1153) |
| < 200 | 19 (50.0) |
| ≧200 | 19 (50.0) |
| Available hospital service |  |
| Emergency department | 34 (89.5) |
| Intensive care unit | 11 (29.0) |
| Infection control department | 34 (89.5) |
| Division of infectious diseases | 14 (36.8) |
| Designated clinic(s) for patients with suspected COVID-19 | 26 (68.4) |

**NOTE.** Data are presented as a number (%) unless otherwise specified.

Abbreviations: HCP, Health care personnel; COVID-19, coronavirus disease 2019

* Hospital type by function is defined by the Japanese ministry of health, labour and welfare.

<https://www.mhlw.go.jp/english/wp/wp-hw4/dl/health_and_medical_services/P39.pdf>

Appendix 2. Baseline characteristics of healthcare personnel with COVID-19

| Characteristics | N=284 |
| --- | --- |
| Age, year, median (range) | 32 (20-81) |
| Female sex | 211 (74.3) |
| Occupation |  |
| Physician | 76 (26.8) |
| Nurse | 141 (49.6) |
| Caregiver assistant | 22 (7.7) |
| Laboratory technician | 1 (0.4) |
| Radiology technician | 6 (2.1) |
| Physiotherapist | 9 (3.2) |
| Pharmacist | 1 (0.4) |
| Administrative personnel | 14 (4.9) |
| Others | 14 (4.9) |
| Involved in direct patient care for patients with COVID-19 | 155 (54.6) |
| Mode of transmission of COVID-19 reported by questionnaire respondents |  |
| Community acquisition | 42 (14.8) |
| Acquisition from family members | 13 (4.6) |
| In-facility (nosocomial) transmission from infected hospitalized patients or infected colleague | 192 (67.6) |
| Unknown | 37 (13.0) |
| Disposition after COVID-19 diagnosis |  |
| Home quarantine | 71 (25.0) |
| Hospitalization in medical ward | 183 (64.4) |
| Hospitalization in the intensive care unit | 0 |
| Designated housing facility | 30 (10.6) |
| Outcome |  |
| Cure and return to work | 267 (94.0) |
| Cure and suspension from work | 8 (2.8) |
| Cure and retirement | 4 (1.4) |
| Hospitalization or home quarantine at the time of survey | 6 (2.1) |
| Death | 0 |

**NOTE.** Data are presented as a number (%) unless otherwise specified.

Abbreviations: HCP, Health care personnel; COVID-19, coronavirus disease 2019

Appendix 3. Open-ended narrative replies from respondents

| Contents | N=38 |
| --- | --- |
| Disruptive actions by other hospitals (e.g., refusal of patient transfer) | 8 (21.1) |
| Children of affected HCP barred from entering nurseries or schools. | 6 (15.8) |
| Children of affected HCP bullied at daycare centers or schools | 4 (10.5) |
| Affected HCP bullied by neighbors | 2 (5.3) |
| Refusal of employing hospital to allow HCP to continue work | 2 (5.3) |
| Refusal of employers to allow family members of affected HCP to work | 2 (5.3) |
| Anonymous nuisance calls | 2 (5.3) |
| Refusal to allow locum tenens to work at hospital | 2 (5.3) |
| Corona Extra® sent to hospital anonymously | 1 (2.6) |
| Inquiries about contracting COVID-19 from HCP aimed at eliciting personal information about affected HCP | 1 (2.6) |
| Physical assaults on infected HCP | 1 (2.6) |
| Mental stress caused by fear of transmitting virus | 1 (2.6) |
| Mobbing by media reporters at hospital entrance | 1 (2.6) |

**NOTE:** Data are presented as a number (%) unless otherwise specified.

Abbreviation: HCP, health care personnel; COVID-19, coronavirus disease 2019
